# A Clinical Study of Photodynamic Therapy for Superficial Esophageal Carcinoma by YAG-OPO Laser

**DOI:** 10.1155/DTE.4.173

**Published:** 1998

**Authors:** Kazunari Yoshida, Shigeru Suzuki, Seishiro Mimura, Hiroyuki Narahara, Hiroshi Tanimura, Yugo Nagai, Kaichi Isono, Teruo Kozu, Hisayuki Fukutomi, Akira Nakahara, Hiromasa Kashimura, Toshio Hirashima, Yoko Murata, Hiroko Ide, Harubumi Kato

**Affiliations:** 1 Department of Gastrointestinal Endoscopy Tokyo Women's Medical College 8-1 Kawada-cho Shinjuku-ku Tokyo 162 Japan; 2 Department of Gastroenterology Osaka Medical Center for Cancer and Cardiovascular Diseases Japan; 3 Second Department of Surgery Wakayama Medical College Japan; 4 Second Department of Surgery Japan; 5 Department of Endoscopic Diagnostics and Therapeutics Chiba University Japan; 6 Department of Internal Medicine University of Tsukuba Japan; 7 Department of Gastroenterology Chiba Nishi General Hospital Japan; 8 Department of Gastroenterological Surgery Tokyo Women's Medical College Japan; 9 First Department of Surgery Tokyo Medical College Japan

## Abstract

A cooperative clinical study of photodynamic therapy (PDT) for superficial esophageal carcinoma was conducted at 6 medical institution. PHE (2mg/kg) with high tumor affinity was used as the oncotropic compound. The light source was a pulse wave YAG-OPO laser with high penetration into the tissue. Irradiation was performed at an energy density of 60–180 J/cm^2^ 48–72 h after PHE administration. Eight lesions in 6 patients were treated. All were type 0-II superficial carcinomas. The depth of invasion was EP–MM for 6 lesions and SM for 2 lesions. A complete response (CR) was achieved in all patients after one session of PDT. Five adverse events, including anemia and fever, were reported by 4 patients, but all were WHO grade 2 or lower and transient. PDT using PHE and YAG-OPO laser was therefore considered effective as a curative therapy for superficial esophageal carcinoma.


*Diagnostic and Therapeutic Endoscopy*, Vol. 4, pp. 173–176
Reprints available directly from the publisher
Photocopying permitted by license only

© 1998 OPA (Overseas Publishers Association)
Amsterdam B.V. Published under license
under the Harwood Academic Publishers imprint,
part of The Gordon and Breach Publishing Group.
Printed in Singapore.

### A Clinical Study of Photodynamic Therapy for Superficial Esophageal Carcinoma by YAG-OPO Laser

KAZUNARI YOSHIDA[a,*], SHIGERU SUZUKI[a], SEISHIRO MIMURA[b], HIROYUKI NARAHARA[b], HIROSHI TANIMURA[c], YUGO NAGAI[c], KAICHI ISONO[d], TERUO KOZU[e], HISAYUKI FUKUTOMI[f], AKIRA NAKAHARA[f], HIROMASA KASHIMURA[f], TOSHIO HIRASHIMA[g], YOKO MURATA[a], HIROKO IDE[h] and HARUBUMI KATO[i]

[a]*Department of Gastrointestinal Endoscopy, Tokyo Women's Medical College, 8-1 Kawada-cho, Shinjuku-ku, Tokyo 162, Japan;* [b]*Department of Gastroenterology, Osaka Medical Center for Cancer and Cardiovascular Diseases;* [c]*Second Department of Surgery, Wakayama Medical College;* [d]*Second Department of Surgery,* [e]*Department of Endoscopic Diagnostics and Therapeutics, Chiba University;* [f]*Department of Internal Medicine, University of Tsukuba;* [g]*Department of Gastroenterology, Chiba Nishi General Hospital;* [h]*Department of Gastroenterological Surgery, Tokyo Women's Medical College;* [i]*First Department of Surgery, Tokyo Medical College*

*(Received 16 May 1997; In final form 15 October 1997)*

**A cooperative clinical study of photodynamic therapy (PDT) for superficial esophageal carcinoma was conducted at 6 medical institution. PHE (2 mg/kg) with high tumor affinity was used as the oncotropic compound. The light source was a pulse wave YAG-OPO laser with high penetration into the tissue. Irradiation was performed at an energy density of 60–180 J/cm$^2$ 48–72 h after PHE administration. Eight lesions in 6 patients were treated. All were type 0-II superficial carcinomas. The depth of invasion was EP–MM for 6 lesions and SM for 2 lesions. A complete response (CR) was achieved in all patients after one session of PDT. Five adverse events, including anemia and fever, were reported by 4 patients, but all were WHO grade 2 or lower and transient. PDT using PHE and YAG-OPO laser was therefore considered effective as a curative therapy for superficial esophageal carcinoma.**

*Keywords:* Endoscopic therapy, Photodynamic therapy, YAG-OPO laser, PHE, Superficial esophageal carcinoma

### INTRODUCTION

Photodynamic therapy (PDT) is performed by applying laser as excitation light 48–72 h after intravenous injection of a photosensitive compound to destroy tumors selectively using the difference in concentration between the tumor and normal tissue. Hayata *et al.* [1] successfully used PDT under endoscopic guidance to treat lung carcinoma, and since then PDT has been applied

* Corresponding author.

to various types of cancers. Okushima *et al.* [2] and Mimura *et al.* [3] achieved a complete response with PDT for superficial esophageal carcinoma. These studies used a continuous-wave Argon-dye laser. In 1990, an Excimer-dye pulse laser was developed. Pulse lasers provide superior penetration of light into tissue and produce the same results using less energy than continuous lasers [4,5]. In a phase III clinical study by the authors, PHE (porfimer sodium) and Excimer-dye laser were found to be effective in the treatment of superficial esophageal carcinoma [6]. However, there was a need for a compact, easy-maintenance laser unit that could be tuned according to the type of photosensitive compound used. This led to the development of a laser for PDT using an Nd:YAG laser excitation optical parametric oscillator (YAG-OPO laser). We report on a clinical study of PDT for superficial esophageal carcinoma using a YAG-OPO laser and PHE.

### MATERIALS AND METHODS

### 1. Participating Institution and Study Period

The present study was conducted at 6 institutions on patients enrolled between June 1995 and December 1996.

### 2. Subjects

The subjects enrolled in the present study were patients with superficial esophageal carcinoma who met the following criteria. (1) A presumptive diagnosis of superficial esophageal carcinoma was made by endoscopy and a definitive diagnosis was made histologically. (2) Radical therapy such as surgery was infeasible. (3) There was no concomitant active carcinoma at any site other than the esophagus. (4) The tumor measured 3 cm or less along the major axis (or had an area of 7 $cm^2$ or less). (5) The whole aspect of the tumor could be observed endoscopically and exposed to laser. (6) The lesion targeted in the present study had not been treated previously. Patients in whom a cancer remained after local endoscopic therapy other than PDT were considered eligible. Before commencement of treatment, written informed consent was obtained from the patient or a family member.

### 3. Patient Characteristics (Table I)

Six patients (8 lesions) were enrolled, all of whom were considered eligible. The mean age was 67.7 years (41–80 years). Five patients had a PS of 0 and one had a PS of 1. All lesions were classified as superficial carcinomas based on tissue type and type 0-II based on endoscopic type. Two lesions showed SM invasion and the others were M carcinomas of EP–MM. Two patients had double lesions, which were considered to be two separate lesions and were treated separately with PDT.

TABLE I Patient characteristics

| | | |
|---|---|---|
| Age | Mean 67.7 | |
| Sex | Male | 5 cases |
| | Female | 1 |
| PS | 0 | 5 cases |
| | 1 | 1 |
| Site | Ce | 1 lesion |
| | Im | 6 |
| | Ei | 1 |
| Tissue type | Wel. dif. SCC | 2 lesion |
| | Mod. dif. SCC | 6 |
| Macroscopic classification | 0-IIb | 3 lesion |
| | 0-IIc | 5 |
| Tumor size (major and minor axis $cm^2$) | | |
| | –1.0 | 2 lesion |
| | 1.1–2.0 | 2 |
| | 2.1–4.0 | 2 |
| | 4.1– | 2 |
| Depth of invasion | EP–MM | 6 lesion |
| | SM | 2 |

PS: performance status; Ce: cervical esophagus; Im: middle thoracic esophagus; Ei: lower thoracic esophagus; EP: intra-epithelium; MM: up to the level of muscularis mucosae; SM: involving submucosa.

### 4. Administration of Agents

PHE (porfimer sodium) is a photosensitive compound (Lederle Japan, containing dihematoporphyrin ester/ether as an active ingredient). PHE 2.0 mg/kg was injected intravenously.

### 5. Laser Irradiation

The laser used in this present study was YAG-OPO laser (Ishikawajima-Harima Heavy Industries Co., Ltd, model iLS-TL-50A). The unit reconverts the wavelengths of a third harmonic generation of 355 nm wavelength converted from the *Q*-switched Nd : YAG laser of 1,064 nm wavelength with exciting an optical parametric oscillator and has the following specifications: (a) wavelength range: 620–670 nm, tuned to 630 nm in this trial; (b) pulse rate: 25 and 50 Hz; (c) maximum pulse energy: 6 mJ/pulse; and (d) pulse width: 5–8 ns.

The laser beam was applied to lesions under direct endoscopic guidance 48–72 h after PHE administration. The irradiation energy density was generally 60–180 J/cm$^2$.

### 6. Management of Patients after PHE Administration

The patients were protected from light after administration of PHE to prevent development of photohypersensitivity.

### 7. Assessment of Efficacy

Endoscopy and histologic biopsy of the lesions were performed at appropriate intervals after laser irradiation to assess the direct efficacy of PDT. Assessment was made using the Criteria for Handling of Esophageal Carcinoma [7]. But patients in whom biopsies remained carcinoma-negative for 4 weeks or longer were considered complete responders.

### 8. Assessment of Safety

Safety was assessed using the descriptions of side effects in the criteria for the assessment of the efficacy of chemotherapy for solid cancers.

### RESULTS

### 1. Antitumor Efficacy (Table II, Fig. 1)

All lesions showed CR after one session of PDT, giving a CR rate of 100%.

TABLE II Clinical response

| CR | PR | NC | CRrate |
|---|---|---|---|
| 8 lesion | 0 | 0 | 100.0% |

CR: complete reaction; PR: partial reaction; NC: no change.

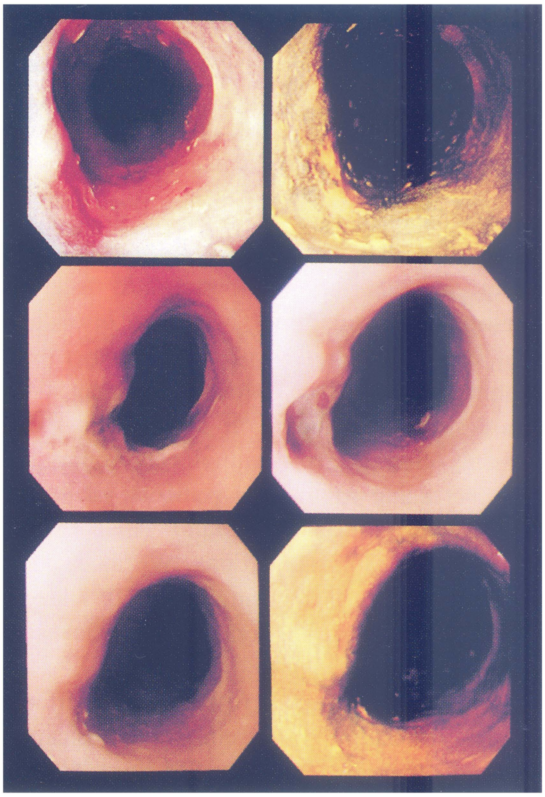

| | |
|---|---|
| Normal picture pre-treatment | Pre-treatment Lugol staining |
| After 1 week | After 2 weeks |
| Normal picture after 1 month | Lugol staining after 1 month |

FIGURE 1 Clinical course of PDT (endoscopic picture). (1) normal picture pre-treatment; (2) pre-treatment Lugol staining; (3) one week later; (4) two weeks later; (5) normal picture one month later; (6) Lugol staining one month later.

TABLE III Side effects

| Side effects | Grade | | | | Total |
|---|---|---|---|---|---|
| | 1 | 2 | 3 | 4 | |
| Anemia | 1 | | | | 1 |
| Renal impairment | 2 | | | | 2 |
| Fever | | 1 | | | 1 |
| Abdominal pain | | 1 | | | 1 |
| Diarrhea | 1 | | | | 1 |

### 2. Safety (Table III)

Four of the six patients reported 5 adverse events thought to be attributable to therapy. All were mild (Grade 2 or less) and disappeared during follow-up, except for 1 patient who was given antipyretics for fever.

### DISCUSSION

Surgical intervention is the standard treatment for esophageal carcinoma, which is often associated with lympho-vascular involvement and lymph node metastasis. However, esophageal carcinomas are more common among the elderly than are other gastrointestinal carcinomas, and in an increasing number of patients, major surgery such as thoracotomy and laparotomy is infeasible due to complications. We assessed the clinical efficacy of PDT using YAG-OPO laser as local therapy for such inoperable superficial esophageal carcinomas.

PDT has been reported to be a highly effective treatment for esophageal carcinoma that is capable of curing carcinomas up to the SM stage [8]. However, SM esophageal carcinomas are frequently associated with lympho-vascular involvement and lymph node metastasis [9]. M carcinomas of EP–MM should therefore be treated using local therapy such as PDT. Although endoscopic mucosectomy is becoming the standard treatment for these M carcinomas, PDT has several advantages in that it is simpler to perform than mucosectomy and can be used in patients prone to hemorrhage or with carcinoma complicated by esophageal varices. Another great advantage of PDT is that it is aimed at inoperable cases and is therefore applicable to SM carcinomas.

The local CR rate of 100% obtained in the present study is higher than the efficacy rates reported previously [2,3,8] and is superior to the efficacy of pulse-wave Excimer-dye laser [6,10]. The laser used in the present study is expected to be highly useful as assessment of safety found no serious side effects in patients with complicating illness.

It was concluded from these results that PDT using the YAG-OPO pulse-wave laser is an effective local therapy.

### *References*

[1] Hayata, Y., Kato, H., Konaka, C. *et al.* Hematoporphyrin derivative and laser photoradiation in the treatment of lung cancer. *Chest* 1982; **81**: 269–277.

[2] Okushima, N., Ide, H. and Habu, F: Laser therapy for esophageal carcinoma. *Therapeutics* 1986; **17**: 160–163.

[3] Mimura, S., Ichii, M., Imanishi, K. *et al.* The application and limitations of HpD photodynamic therapy in esophageal and gastric carcinomas. *Cancer and Chemotherapy* 1988; **15**: 1440–1444.

[4] Sakai, H., Aizawa, K., Kato, H. *et al.* Photodynamic therapy using an Excimer-dye laser. *Journal of the Japanese Society for Laser Medicine* 1986; **6**: 99–102.

[5] Okunaka, T., Kato, H., Konaka, C. *et al.* A comparison between Argon-dye and Excimer-dye laser for photodynamic effect in transplanted mouse tumor, *Jpn. J. Cancer Res.* 1992; **83**: 226–231.

[6] Yoshida, K., Suzuki, S., Mimura, S. *et al.* A phase III clinical study of photodynamic therapy using PHE and an Excimer-dye laser for superficial esophageal carcinoma. *Cancer and Chemotherapy* 1993; **20**: 2063–2066.

[7] Ed. by Study Group on Esophageal Illness: Criteria for Handling of Esophageal Carcinoma (8th ed.), Kanehara Shuppan, 1992.

[8] Okushima, N. A study of endoscopic laser photochemical therapy for superficial esophageal carcinoma. *Gastroenterol. Endosc.* 1987; **29**: 1105–1114.

[9] Ide, H., Yoshida, K. and Murata, Y: Progress and therapeutic problems in the diagnosis of esophageal carcinoma. Nihon Medical News. No. 3507, 1991.

[10] Furuse, K., Kodama, N., Kubota, H. *et al.* A phase III clinical study of photodynamic therapy using Excimer-dye laser combined with PHE in early lung, gastric and esophageal cancers. *Journal of Japanese Society for Laser Medicine* 1993; **14**: 9–15.

